# Global Warming and Mass Mortalities of Benthic Invertebrates in the Mediterranean Sea

**DOI:** 10.1371/journal.pone.0115655

**Published:** 2014-12-23

**Authors:** Irene Rivetti, Simonetta Fraschetti, Piero Lionello, Enrico Zambianchi, Ferdinando Boero

**Affiliations:** 1 Dipartimento di Scienze e Tecnologie Biologiche ed Ambientali, Università del Salento, CoNISMa, Lecce, Italy; 2 CMCC Euro-Mediterranean Center on Climate Change, Lecce, Italy; 3 Dipartimento di Scienze per l′Ambiente, Università degli Studi di Napoli “Parthenope”, CoNISMa, Napoli, Italy; 4 Istituto di Scienze Marine del CNR, Genova, Italy; Università della Calabria, Italy

## Abstract

Satellite data show a steady increase, in the last decades, of the surface temperature (upper few millimetres of the water surface) of the Mediterranean Sea. Reports of mass mortalities of benthic marine invertebrates increased in the same period. Some local studies interpreted the two phenomena in a cause-effect fashion. However, a basin-wide picture of temperature changes combined with a systematic assessment on invertebrate mass mortalities was still lacking. Both the thermal structure of the water column in the Mediterranean Sea over the period 1945–2011 and all documented invertebrate mass mortality events in the basin are analysed to ascertain if: 1- documented mass mortalities occurred under conditions of positive temperature trends at basin scale, and 2- atypical thermal conditions were registered at the smaller spatial and temporal scale of mass mortality events. The thermal structure of the shallow water column over the last 67 years was reconstructed using data from three public sources: MEDAR-MEDATLAS, World Ocean Database, MFS-VOS programme. A review of the mass mortality events of benthic invertebrates at Mediterranean scale was also carried out. The analysis of *in situ* temperature profiles shows that the Mediterranean Sea changed in a non-homogeneous fashion. The frequency of mass mortalities is increasing. The areas subjected to these events correspond to positive thermal anomalies. Statistically significant temperature trends in the upper layers of the Mediterranean Sea show an increase of up to 0.07°C/yr for a large fraction of the basin. Mass mortalities are consistent with both the temperature increase at basin scale and the thermal changes at local scale, up to 5.2°C. Our research supports the existence of a causal link between positive thermal anomalies and observed invertebrate mass mortalities in the Mediterranean Sea, invoking focused mitigation initiatives in sensitive areas.

## Introduction

Global environmental change is a major threat to marine ecosystems. Changes in Sea Surface Temperature (SST) are acknowledged as an important component of global change [1] and have long been charged as one of the main physical drivers influencing both marine biodiversity and the services it provides. Ultimate direct or indirect consequences of thermal anomalies include alterations in the patterns of distribution of species and assemblages [2]–[4], species' phenology [5], spreads in both pathogens and invasive species, and mass mortalities [6]–[9]. Rises in SST are associated with modifications of the vertical thermal structure of the upper ocean, with possible bio-ecological effects that are not limited to the first meters of the water column [10], [11]. However, experimental simulations of the effects of thermal anomalies along the water column on marine ecosystems can be complex [12]–[14] and hydrological measurements are seldom collected consistently in space and time with the specific goal of documenting the effects of warming on marine ecosystems [15]. Expanding present knowledge on the magnitude of changes of the SST to the upper water column (0–50 meters) combining data at wide temporal and spatial scale is critical to anticipate future changes, propose mitigation strategies, and set conservation priorities.

Due to its relatively small volume, the Mediterranean Sea is expected to react faster to global change than the open ocean [16], [17] and is one of the areas where important biotic changes, linked to abiotic changes, have been already extensively documented [18]–[20]. Recent studies showed that 20% of the entire basin and 60–99% of the territorial waters of EU member states are heavily impacted. Human impacts are high in all ecoregions and territorial waters, resulting from multiple drivers, rather than single individual uses or stressors, with climatic drivers (increasing temperature and UV, and acidification), demersal fishing, ship traffic, and, in coastal areas, pollution from land accounting for most cumulative impacts [21]–[23].

Long-term change is usually ascribed to three main physical processes: 1- basin scale increase of SST, documented by satellite data, since the 1980s [24], [25]; 2- temperature and salinity increases of Western Mediterranean deep-water (below 600 m), since the 1950s [26]–[29]; 3- salinity increase and temperature decrease in the Levantine Intermediate Water (LIW), since the 1950s [30]–[32]. However, observations are very irregular in space and time and trends critically depend on both the covered time frame and the employed statistical approach [33].

In the Mediterranean Sea, together with the establishment of species of tropical affinity [17], several episodic events of mass mortalities affecting species of cold-water affinity have been extensively documented starting from 1983 and reported with increasing frequency since 1992 [20], [34], [35]. Coma et al. [34] showed that changes in the stratification of the water column at a station located 1.7 km offshore of the Medes islands (North-West Mediterranean) produced a 40% lengthening of summer conditions culminating in mass mortality events of invertebrates (mainly gorgonians and sponges), with a biomass loss of>35%. Crisci et al. [35] considered inter-regional and annual differences in temperature conditions associated with mass mortality events by analysing 8-year long high-resolution temperature time series (1999–2006) in four regions of the North-West Mediterranean Sea with differing hydrological conditions. The bio-ecological consequences of increasing temperature are large, as witnessed by increasing reports on disease outbreaks and mass mortalities affecting temperate sessile invertebrates such as sponges and cnidarians, long suspected of being particularly sensitive to temperature increases [36], [37].

To date, studies considering changes of temperature from SST to the first 50 m of the water column at the scale of the whole Mediterranean Sea are extremely limited [32], [29], and none matched this information with the occurrence of biological events possibly connected to thermal anomalies. Our study further expands on this issue by: 1- quantifying how the documented increases in SST, recorded over the last years across the Mediterranean Sea affected the thermal structure of the water column; 2- assessing if atypical thermal conditions events have been systematically registered at the smaller spatial and temporal scales of mass mortalities. To answer these questions, the thermal structure of the uppermost part of the water column (0–50 meters) was analysed both at the spatial scale of the whole Mediterranean Sea (thousands of kilometres) and at the smaller spatial scale of mass mortality events documented in the literature (hundreds of meters), by using in *situ* vertical profiles of temperature collected over the last 67 years. All hydrographic temperature profiles from available databases were combined so as to produce a global picture of temperature changes across the basin. More specifically, to identify climate change signals in the upper water column, possibly connected to biological changes, basin scale trends in temperature were analysed at 0–10 m, 11–30 m, 31–50 m depth layers for the period 1945–2011, in the months in which mass mortalities have been reported. All records of invertebrate mass mortalities in the Mediterranean Sea were reviewed. Temperature data were mapped together with the occurrence of mass mortalities, to assess the spatial overlap between the occurrence of these biological events and thermal trends. Finally, in those areas where mass mortalities were recorded, the temperature profiles have been analysed to assess the temperature behaviour along the water column during mass mortality events, compared to the period when mass mortalities where never observed.

This is the first time that thermal change is analysed at this temporal and spatial scale in the Mediterranean, linking it to biological responses, showing how global change can affect the biota at basin scale.

## Materials and Methods

### Description of temperature data

The temperature profiles contained in MEDAR/MEDATLAS [38], the largest historical database of hydrographic variables collected in the Mediterranean and Black Sea, are the main source of data in this study. Data range from 1889 to 2000, and were collected with bottles, Mechanical Bathy-Thermographs (MBT), eXpendable Bathy-Thermographs (XBT), and Conductivity-Temperature-Depth (CTD). MEDAR/MEDATLAS profiles were integrated with two other datasets: CTD and XBT data (covering the period 2000–2013) of the *World Ocean Database 2013* (WOD13) [39] and XBT measurements (covering the period 1999–2011) of the Mediterranean Forecasting System-Voluntary Observing Ship program (MFS-VOS) [40].

Our analyses focused on the period 1945–2011 because before 1945 and after 2011 data have not been systematically collected or deposited. The months included in the analyses were July, August, September, October and November, when thermal stratification is most marked. The appraisal of the distribution of temperature along the water column and its evolution in time requires a careful quality control of individual profiles fully described in S1 Appendix (see also S1 Fig.). Out of 115,619 analysed temperature profiles available in the period 1945–2011 and in the selected months, 17,688 were discarded after the quality control. The remaining 97,931 profiles were linearly interpolated at 1 m intervals in depth before being analysed. S2 and S3 Figs. show their temporal and spatial distribution. The largest number of temperature profiles refers to the period between 1965 and 1995. Most information about temperature in the Mediterranean Sea come from MBT and XBT data, inherently less accurate than CTD data, which are available since 1975 and represent ∼13% of the data used in this study. S2 Fig. shows the temporal distribution of the observations. S3 Fig. shows data density in the whole investigated period (1945–2011), using cells of 0.5° latitude by 0.5° longitude for July, August, September, October and November. Data distribution is inhomogeneous, with no observations along the coasts of Tunisia and Libya. The highest densities of observations for all considered months cover the gulf of Lyons, the Ligurian Sea, the North Adriatic Sea and the Alboran Sea.

### Review on mass mortalities of marine benthic invertebrates

During the last 30 years, the increasing number of mass mortality events affecting marine invertebrates in the Mediterranean Sea called for the identification of common features and possible causes. In this framework, an extensive search of all data-bases in ISI Web of Knowledge, from 1945 to 2011, was carried out to document studies on mass mortalities (S2 Appendix). A specific search was carried out within the “Topic” field, with a factorial combination of the keywords “Mediterranean” and “mortality” alone, and together with “mass”. The geographical coordinates of the event, the species involved, the response variable, or metric, used to assess the response to the event, the starting date of the event, the depths at which signs of stress/mortality have been detected, the intensity of the effects (assessed either as low, medium, high, when only qualitative information is given, or as percentage of affected population/colony), the potential synergy with other threats have been documented for each study (S1 Table). Where appropriate, the authors were contacted for additional information.

### Combining physical data to mass mortalities

**Figure 1:**
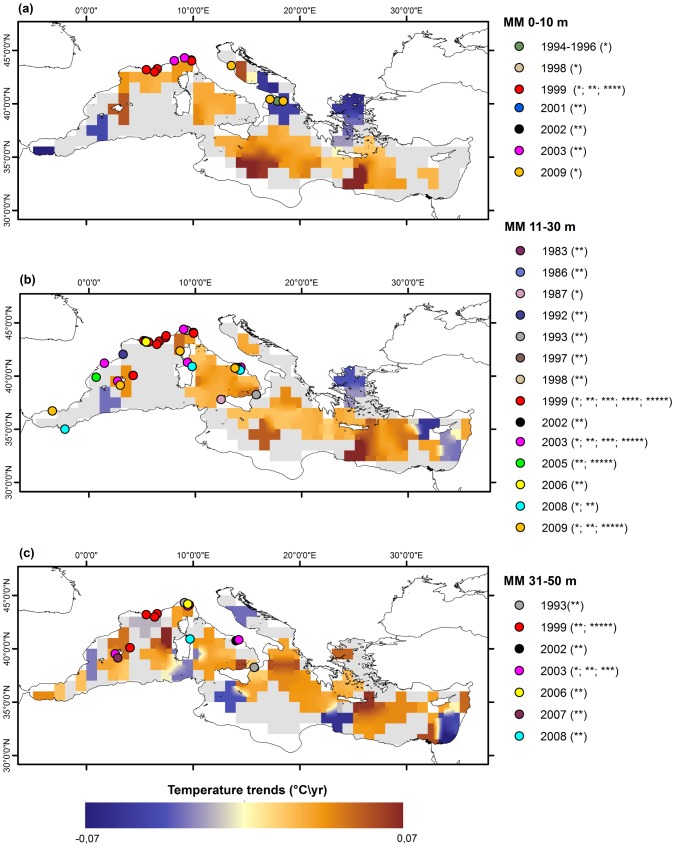
Temperature trends at Mediterranean basin scale. Temperature trends at 0–10 m (a), 11–30 m (b), 31–50 m (c) depth layers for the period 1945–2011 in July-November. Linear regressions have been calculated on grids of 1° latitude by 1° longitude and tested for statistical significance at the 90%. Significant increased/decreased temperature trends are reported as coloured cells, not significant increased/decreased temperature trends are reported as grey areas. Dots refers to the locations of documented mass mortalities for depth layer, each colour represents a single event. The asterisks in the legend of mass mortalities (MM) events refer to the taxa affected: * stands for sponges, ** for cnidarians, *** for bryozoans, **** for ascidians, ***** for bivalves.

**Figure 2:**
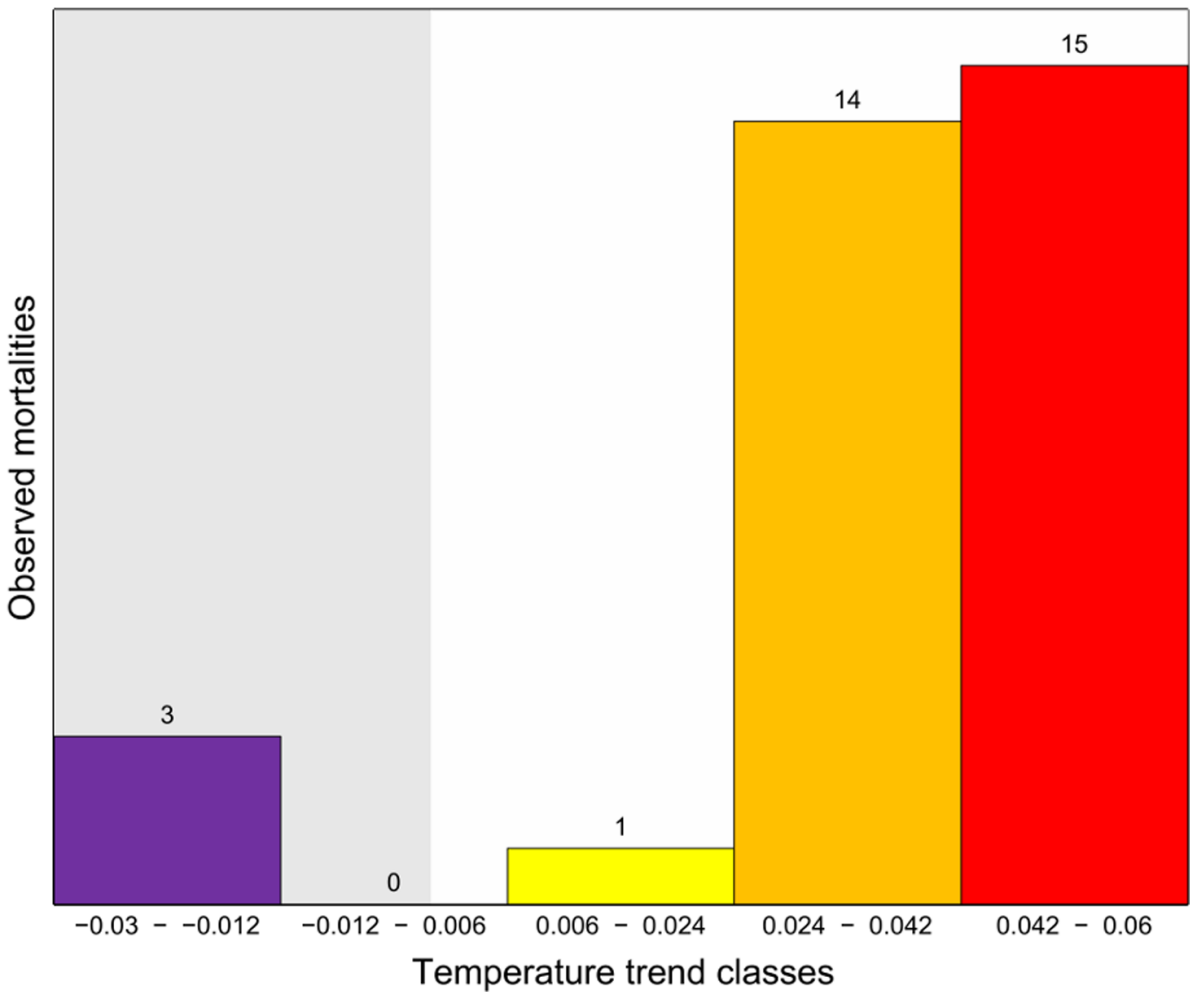
Distribution of observed mortalities over temperature trend intervals. Temperature trend is measured in °C/yr. The grey background corresponds to a negative temperature trend, while the white background corresponds to a positive one.

Data have been spatially and temporally organized by using two approaches. First, to provide a global picture of temperature changes across the Mediterranean Sea, linear temperature trends over the period 1945–2011 have been mapped, focusing on months when mass mortalities occurred (from July to November) and on three depth ranges: 0–10 m, 11–30 m, 31–50 m (Fig. 1). The analysis considered linear regressions on yearly basis, on grids of 1° latitude by 1° longitude. For each box and depth layer, linear regressions have been tested for statistical significance at the 90% and only significant values have been plotted as coloured cells, not significant values have been plotted as gray cells. The spatial distribution of mass mortalities has been overlapped to the map representing temperature trends for the three depth ranges, to assess the spatial overlap between the occurrence of these biological events and thermal trends (Fig. 1). In addition to the analyses of the qualitative spatial overlap of the two data sets shown in Fig. 1, the distance between their empirical distributions was first assessed simply by comparing the occurrences of mortality events in areas with negative temperature trends with those in areas with positive temperature trends; thereafter, the relationship between occurrences and temperature trends was further assessed through the Kolmogorov-Smirnov test (e.g. [41]). The test was performed over each portion of the water column shown in Fig. 1 (i.e. 0–10 m, 11–30 m, 31–50 m), as well as for the entire 0–50 m depth layer. Given the paucity of data in individual layers, only results relative to the whole 0–50 m portion of the water column will be presented in this paper (Fig. 2, S2 Table). We considered the mortalities that occurred in areas where the trend was assessed with a 90% statistical significance, even though, for the sake of completeness, the test was run also over all observed mortality data, yielding the same results.

**Figure 3:**
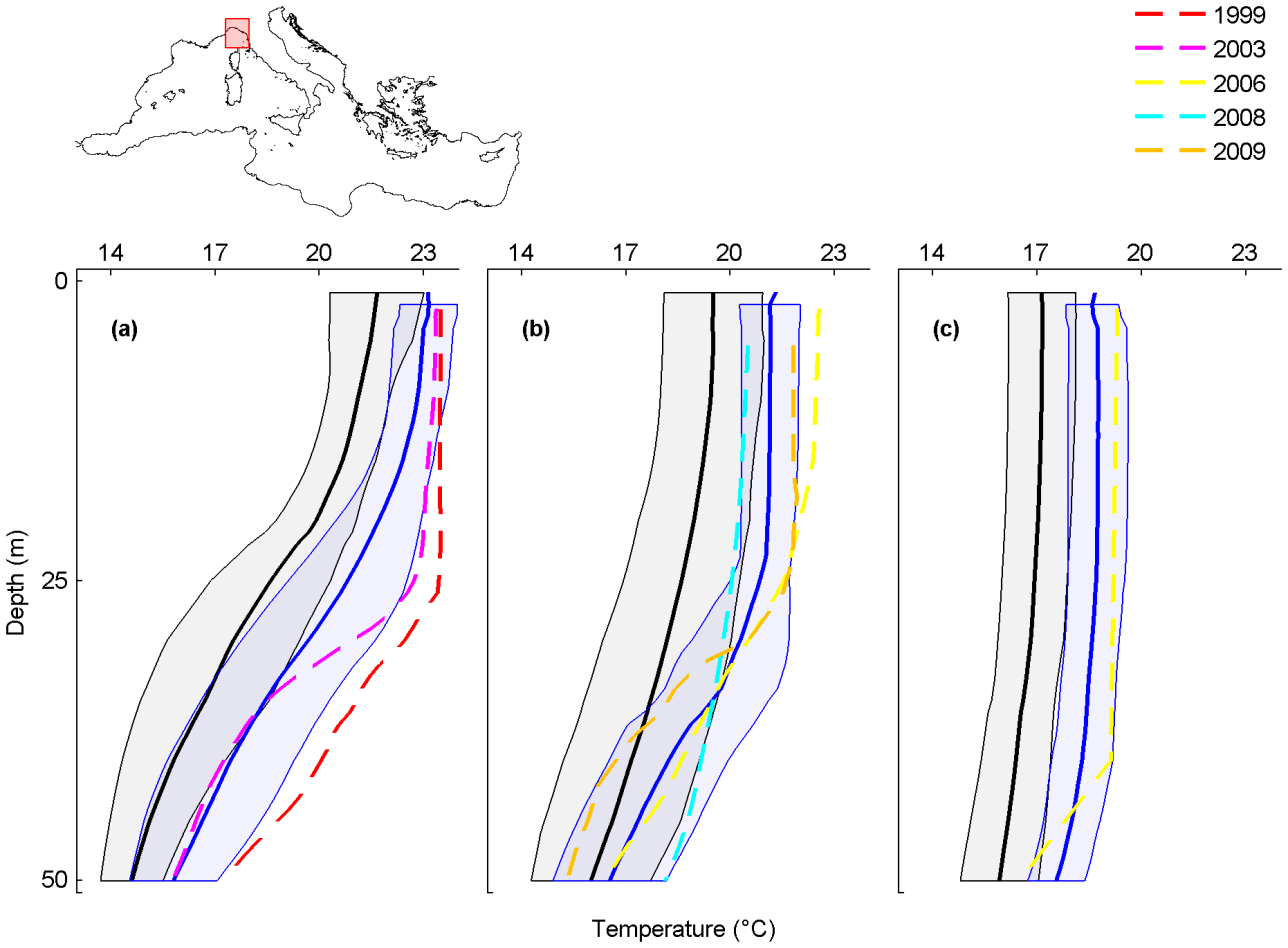
Monthly average temperature profiles and mass mortality in the Ligurian Sea. Analysed months are September (a), October (b) and November (c). Black and blue lines are the monthly-averaged temperature profiles for the periods 1945–1982 and 1983–2011, shaded areas are their standard deviations. The dashed lines are the average temperature profiles for September 1999 (red), September 2003 (magenta), October and November 2006 (yellow), October 2008 (cyan) and October 2009 (orange) when mass mortalities occurred.

**Figure 4:**
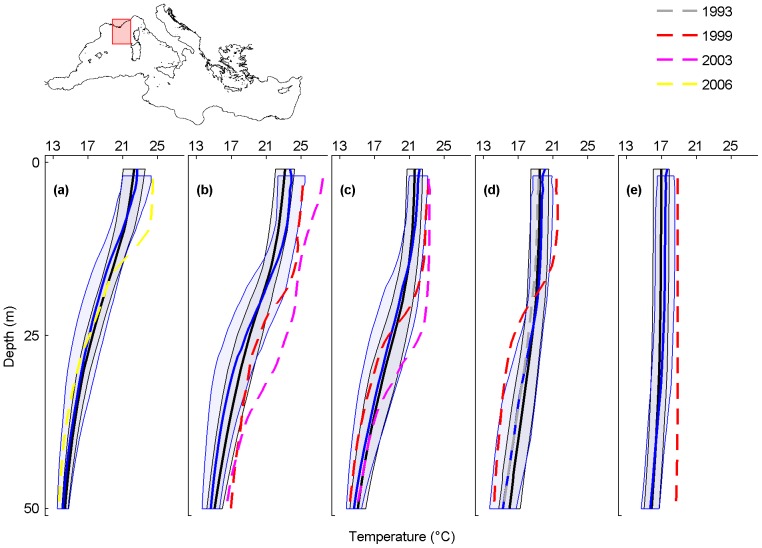
Monthly average temperature profiles and mass mortality in the Provence Coast. Analysed months are July (a), August (b), September (c), October (d) and November (e). Black and blue lines are the monthly-averaged temperature profiles for the periods 1945–1982 and 1983–2011, shaded areas are their standard deviations. The dashed lines are the average temperature profiles for October 1993 (grey), August, September, October and November 1999 (red), August and September 2003 (magenta) and July 2006 (yellow), when mass mortalities occurred.

**Figure 5:**
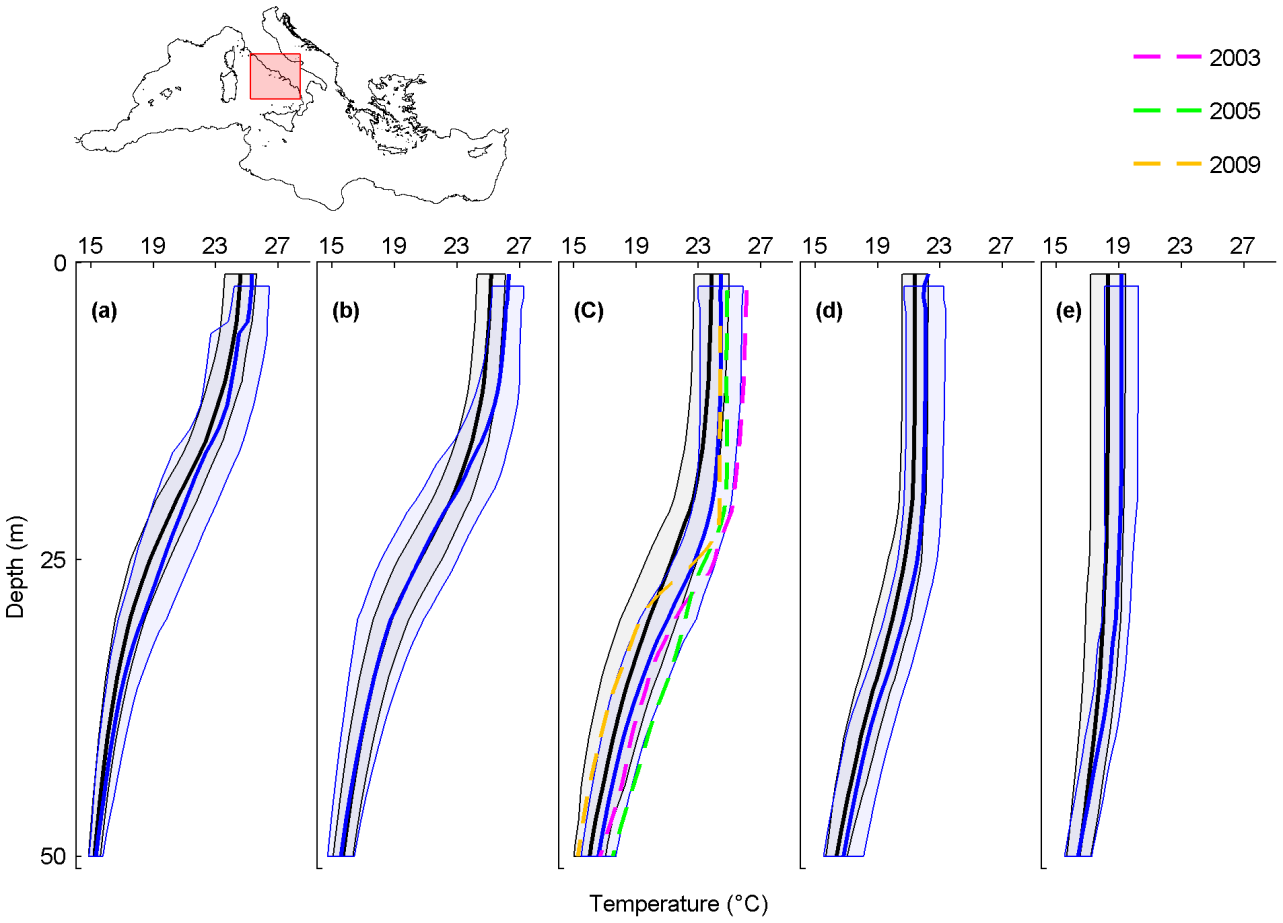
Monthly average temperature profiles and mass mortality in the Eastern Tyrrhenian. Analyzed months are July (a), August (b), September (c), October (d) and November (e). Black and blue lines are the monthly-averaged temperature profiles for the periods 1945–1982 and 1983–2011, shaded areas are their standard deviations. The dashed lines represent the average temperature profile for September 2003 (magenta), September 2005 (green) and September 2009 (orange), when mass mortality occurred.

**Figure 6:**
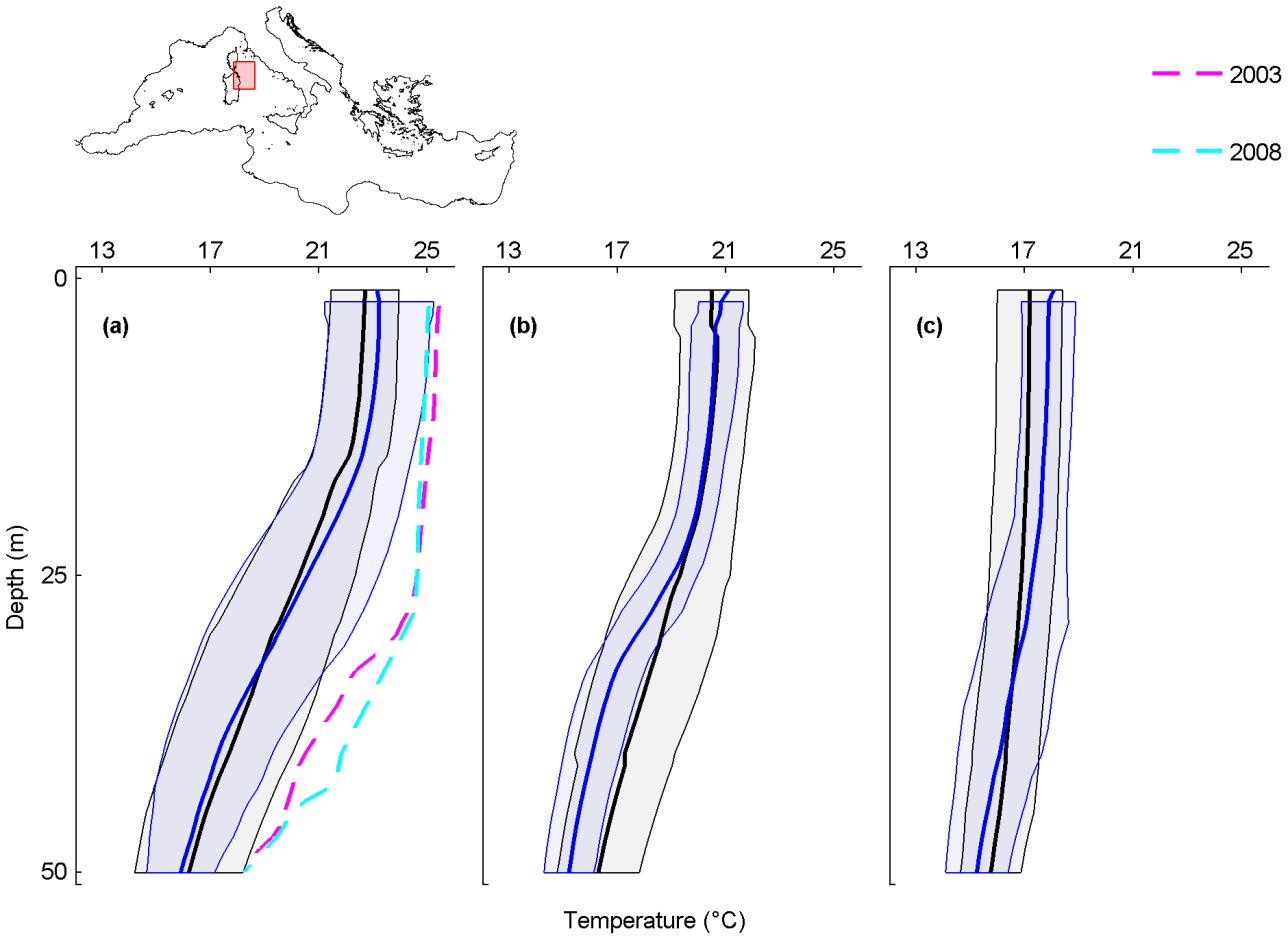
Monthly average temperature profiles and mass mortality in the Western Tyrrhenian. Analyzed months are September (a), October (b) and November (c). Black and blue lines are the monthly-averaged temperature profiles for the periods 1945–1982 and 1983–2011, shaded areas are their standard deviations. The dashed lines represent the average temperature profile for September 2003 (magenta) and September 2008 (cyan), when mass mortality occurred.

**Figure 7:**
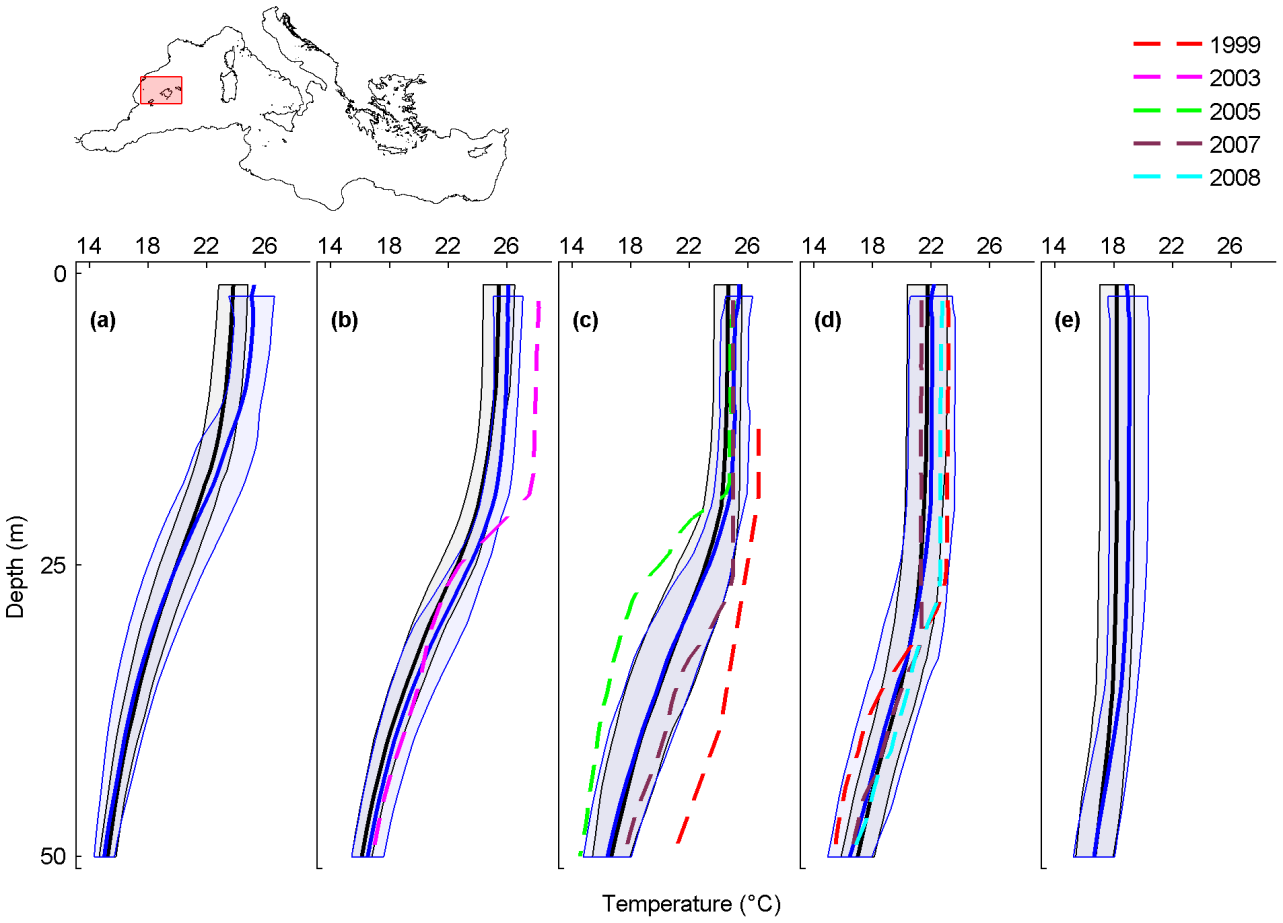
Monthly average temperature profiles and mass mortality in the Balearic and Columbretes islands. Analysed months are July (a), August (b), September (c), October (d) and November (e). Black and blue lines are the monthly-averaged temperature profiles for the periods 1945–1982 and 1983–2011, shaded areas are their standard deviations. The dashed lines represent the average temperature profile for September and October 1999 (red), August 2003 (magenta), September 2005 (green), September and October 2007 (brown) and October 2008 (cyan), when mass mortality occurred.

Second, in those areas where mass mortalities were observed, the monthly temperature profiles available in subareas shallower than 2,000 m have been analysed in boxes with size ∼2° latitude ×∼2° longitude. This analysis compared, for each box, the monthly-averaged temperature profiles for the periods 1945–1982 and 1983–2011, their standard deviations, and the temperature profiles registered in those years and months when mass mortalities were observed (Figs. 3–7). The two periods have been chosen considering that the first documented mass mortality event occurred in 1983 [42]. The study focused on the Ligurian Sea, the Provence Coast, the Eastern and Western Tyrrhenian Sea and the Balearic and Columbretes islands, where mortality events were repeatedly reported. Other areas (e.g. the Alboran Sea) were not included since temperature data were not sufficient to run the analyses. The months included in the analyses were July, August, September, October and November (with the exception of the Ligurian Sea and the Western Tyrrhenian Sea, where data were available only from September to November).

In addition, the monthly frequency of the number of positive temperature extremes compared to the mean temperature and to the mean temperature plus the standard deviation over the period 1945–2011 has been assessed for the periods 1945–1982 and 1983–2011, in all these areas at 0–10 m, 11–30 m and 31–50 m depth for the months July–November (S3 Table).

Temperature datasets concerning coastal areas (shallower than 250 m) were 492 out of 2,365 in the Ligurian Sea, 1,286 out of 8,194 in the Provence Coast, 354 out of 5,442 in the Eastern Tyrrhenian, 232 out of 1,286 in the Western Tyrrhenian and 949 out of 2,324 in the Balearic and Columbretes islands.

## Results

### Mass mortalities events in the Mediterranean Sea

An extended literature analysis yielded 35 publications that report on 19 mass mortality events involving 59 species across the Mediterranean Sea (S1 Table). After the first event, in 1983 in the Ligurian Sea, the frequency of reports of mass mortalities steadily increased since the year 1992. Cnidarians (e.g. *Paramuricea clavata*, *Eunicella singularis*, *Eunicella cavolinii*, *Cladocora caespitosa*) and sponges (e.g. *Spongia officinalis*, *Ircinia* spp.) are the most affected taxa; most mortalities occurred in the first 30 m in late summer-early fall. Mass mortality events have been mostly reported in the Western Mediterranean, with the exceptions of Porto Cesareo (Apulia, Italy) in 1994–1996 [43], the North Aegean Sea in 1999 [44], Cape San Vito and Torre Sant'Andrea (Apulia, Italy) in 2009 [45], the Cornero Promontory (North Adriatic Sea, Italy) in 2009 [46].

The occurrences of documented mass mortalities range from local-scale (several tens of meters or kilometres) to regional-scale (several hundreds of kilometres). The two most dramatic events in terms of geographical extension (1,000 km of coastline) and number of affected species (approximately 30 macro-benthic species including sponges, cnidarians, bivalves, ascidians, and bryozoans) occurred during the summers of 1999 and 2003 along the North Western Mediterranean coasts. These two events coincided with positive thermal anomalies, with temperatures of 3–4°C above average and a prolonged water column stability in late summer [44], [47], [48]. Almost all studies associated mass mortalities to positive thermal anomalies in late summer and early fall, when species experience energetic constraints [34], [35]. However, the same regions affected by mass mortalities in different years exhibited differential responses among species, both in magnitude, ranging from low to high mortality, and in the affected depth range (see S1 Table), thus indicating that factors other than mean temperature may be involved in determining the observed changes.

### Mapping temperature trends and mass mortalities at basin scale

Fig. 1 shows the spatial distribution of linear temperature trends calculated on a yearly basis for the period 1945–2011 in the months July–November at three depth layers. The trends toward increased/decreased temperatures that are significant at the 90% confidence level are reported as coloured cells.

Most coloured cells show trends toward increased temperature; in particular, a warming signal is present in all layers in the Balearic Sea, in the Ligurian Sea, in the Tyrrhenian Sea and from the Ionian Sea to the Nile Delta. On the contrary, intense cooling occurs in the Northern Aegean Sea and in the Southern Adriatic Sea in the uppermost 30 m and 10 m, respectively. Most areas where mass mortalities occurred at depth from 0 to 10 m showed a significant warming, with the exception of the mass mortality events of sponges that occurred in the Southern Adriatic Sea [43], [45]. From 11 to 30 m, the mass mortalities that occurred in the Ligurian Sea, in the Western and Eastern Tyrrhenian Sea and in the Balearic and Columbretes islands match with the warming areas; the matches did not occur for the mass mortalities in the Provence Coast, and in the Catalan and Alboran Seas. From 31 to 50 m, mass mortalities are associated with positive temperature trends, with the exception of the mass mortalities that occurred in the Provence coast, where decreasing temperature trends were registered.

Three mortality events were documented in the 0–50 m layer in areas with a negative temperature trend, whereas thirty mortality events were documented in areas with a positive temperature trend. This 1 to 10 ratio largely exceeds the ratio of areas with a negative temperature trend vs areas with a positive trend, which is slightly less than 1 to 4 (namely 25 to 104), suggesting a significant, unbiased relationship between positive temperature trends and mortality events. The distribution of observed mortalities vs temperature trend, the latter subdivided into 5 evenly sized intervals (Fig. 2), further strengthens this interpretation.

This relationship was also corroborated by the results of a Kolmogorov-Smirnov test, comparing the distributions of observed mortalities with a uniform distribution of occurrences, uniformly distributed over evenly-sized temperature trend intervals. Its results (reported in S2 Table) allowed to exclude with a 99% probability the hypothesis that the distribution of mortalities is independent from the temperature trend, thus supporting the previous qualitative and quantitative outcomes. These results, however, call for some caution for the following reasons: mortalities of benthic organisms have been observed occasionally, and no systematic surveys focused on such events; the singularity of events makes their statistical treatment not straightforward, since mortality occurrences are defined only as positive outcomes, not opposed to negative ones; different organisms may exhibit a different sensitivity (and/or resilience) to ambient temperature changes, as will be discussed in the final section of this paper. However, data and results reported in S2 Table allowed to exclude with a 99% probability the hypothesis that the distribution of mortalities is independent from the temperature trend, thus supporting the previous qualitative and quantitative outcomes.

### Matching temperature profiles with mortality events at local scale

The temperature profiles have been analysed at local scale (about 300 km^2^) in the Ligurian Sea, the Provence Coast, the Western Tyrrhenian, the Eastern Tyrrhenian, and the Balearic and Columbretes islands.

The comparison of the frequencies of positive temperature extremes in the periods 1945–1982 with those of 1983–2011 (departing both from the mean temperature and from the mean temperature plus the standard deviation over the period 1945–2011) shows an increased frequency of positive temperature extremes in the Ligurian Sea for September, October and November, in the Eastern Tyrrhenian Sea, and in the Balearic and Columbretes islands for all considered months. These results further confirm the match between temperature extremes and the occurrence of mass mortalities (S3 Table).

### Ligurian Sea

In the Ligurian Sea, mass mortalities of marine organisms are reported from 1986 to 2009 (S1 Table). Temperature profiles are available for the mortality events of 1999, 2003, 2006, 2008 and 2009.

All 1983–2011 monthly-averaged temperature profiles are shifted towards higher temperatures when compared with the 1945–1982 monthly-averaged temperature profiles, particularly in September and October when warming affected the uppermost 50 m (maximum temperature difference of 2.3°C at 26 m in September and of 2.8°C at 23 m in October) (Fig. 3a,b; black and blue lines). In addition, all temperature profiles registered in the months and years when mortality events occurred (coloured lines) are shifted towards higher temperatures than the 1945–1982 monthly-averaged temperature profiles (black lines). The mortality events of September 1999 and 2003 clearly occurred under conditions of particularly positive thermal anomaly (Fig. 3a). For September 1999, at 26 m, the temperature was 5°C and 2.8°C higher than the 1945–1982 and 1983–2011 monthly-averaged values, respectively. Here, mass mortalities of cnidarians and sponges were observed from the surface to 70 m [49], [44], [50] (S1 Table). In September 2003, at 26 m, the temperature was 4.1°C and 1.9°C higher than the 1945–1982 and the 1983–2011 monthly-averaged values, respectively. Here, mass mortalities of cnidarians and sponges were observed from the surface to 40 m, in coincidence with the positive thermal anomaly [9], [50]–[52] (S1 Table).

### Provence Coast

In the Provence Coast, mass mortalities of marine organisms are reported since 1983 to 2006 (S1 Table). Temperature profiles are available for the mortality events of 1993, 1999, 2003 and 2006.

In August, the 1983–2011 monthly-averaged temperature profile is shifted towards higher temperatures when compared with that of 1945–1982, with a maximum temperature difference of 1°C at 8 m (Fig. 4b; black and blue lines). Monthly temperature profiles registered when mortality events occurred (coloured lines) are all shifted towards higher temperatures than both the 1945–1982 and 1983–2011 monthly-averaged temperature profiles (black and blue lines), with the exception of the mortality event of October 1993, when thermal conditions were not particularly critical (Fig. 4d). As for the Ligurian Sea, the mass mortality events of 1999 and 2003 occurred under conditions of very large positive thermal anomaly. More specifically, in 1999, temperatures were particularly high near the surface in August, September and October (maximum temperature difference between temperature profile registered during the 1999 mortality event and the 1945–1982 monthly-averaged temperature profile of 2.7°C at 14 m in August, of 1.7°C at 14 m in September and of 2.1°C at 8 m in October). In November 1999, a large positive thermal anomaly of ∼2.8°C, if compared with those of 1945–1982 and 1983–2011 monthly-averaged temperature profiles, affected the first 50 m of the water column. Here, damaged colonies of the red coral *Corallium rubrum* were observed up to 26 m [53] (S1 Table). In August and September 2003, mass mortalities of cnidarians, sponges and molluscs were observed [54], [9] (S1 Table) along with large positive thermal anomalies in the first 30 m (maximum temperature difference between temperature profile registered during the 2003 mortality event and the 1983–2011 monthly-averaged temperature profile of 4.8°C at 28 m in August, and of 3.4°C at 24 m in September).

### Eastern Tyrrhenian Sea

In the Eastern Tyrrhenian Sea, mass mortalities of marine organisms are reported from 2002 to 2009 (S1 Table). Temperature profiles are available for the mortality events of 2003, 2005 and 2009.

All 1983–2011 monthly-averaged temperature profiles are shifted towards higher temperatures when compared with those of 1945–1982, particularly in September (maximum temperature difference of 1.5°C at 24 m) (Fig. 5c; black and blue lines). In addition, all temperature profiles registered when mortality events occurred (coloured lines) are shifted towards higher temperatures than the 1945–1982 monthly-averaged temperature profiles (black lines).

As for the Ligurian Sea and the Provence Coast, the mass mortality event of September 2003 clearly occurred under conditions of particularly positive thermal anomaly, the temperature being 2°C higher than the 1945–1982 monthly-averaged temperature profile from the surface up to 20 m. Here, mass mortalities of cnidarians were observed up to 32 m [9] (S1 Table).

### Western Tyrrhenian Sea

In the Western Tyrrhenian Sea, mass mortalities of marine organisms are reported from 2001 to 2008 (S1 Table). Temperature profiles are available only for the mortality events of 2003 and 2008.

Only in September and November the 1983–2011 monthly-averaged temperature profiles are shifted towards higher temperatures when compared to the 1945–1982 monthly-averaged temperature profiles (maximum temperature difference of 0.6°C at 17 m in September and of 0.7°C at 5 m in November) (Fig. 6a,c; black and blue lines). The temperature profiles registered when mortality events occurred (coloured lines) are shifted towards higher values in the whole upper layer than both the 1945–1982 and the 1983–2011 monthly-averaged temperature profiles (black and blue lines).

For September 2003, at 30 m, the temperature was 4.6°C higher than the 1945–1982 monthly-averaged temperature profile and 4.5°C higher than the 1983–2011 monthly-averaged temperature profile. Here, mass mortalities of cnidarians, sponges, bryozoans and molluscs were observed from the surface to 30 m [9] (S1 Table). In September 2008, at 30 m, the temperature was 4.8°C higher than the 1945–1982 monthly-averaged temperature profile and 4.7°C higher than the 1983–2011 monthly-averaged temperature profile. Here, mass mortality of the cnidarian *Paramuricea clavata* was observed up to 35 m [55], [56] (S1 Table).

### Balearic and Columbretes islands

In the Balearic and Columbretes islands, mass mortalities of marine organisms are reported from 1999 to 2009 (S1 Table). Temperature profiles are available for the mortality events of 1999, 2003, 2005, 2007 and 2008.

Only in July, August and November, the 1983–2011 monthly-averaged temperature profiles are shifted towards higher values than those of 1945–1982 (maximum temperature difference of 1.4°C at 4 m in July, of 1°C at 19 m in August and of 0.8°C at 4 m in November) (Fig. 7a,b,e; black and blue lines).

Particularly warm conditions (at the upper limit of interannual variability) have been recorded in August 2003 and in September and October 1999, when mass mortalities occurred. For August 2003, the temperature was 3.3°C higher than the 1945–1982 monthly-averaged temperature profile, and 2.4°C higher than the 1983–2011 monthly-averaged temperature profile. Here, mass mortalities of cnidarians, sponges, and bryozoans were observed from the surface to 40 m [9] (S1 Table). In September 1999, from 30 to 50 m, the temperature was from 4°C to 5.2°C higher than the 1945–1982 and 1983–2011 monthly-averaged temperature profiles. In the same month and depth range, up to 67% of colonies of the cnidarian *Eunicella singularis* were affected by mass mortalities [57] (S1 Table).

The mass mortality event of September 2005 and October 2007 did not occur under atypical warm conditions.

## Discussion

Our results show that positive temperature trends have been observed at most areas where mass mortalities have been reported. In addition, in the periods when mass mortalities have been documented, the monthly average temperature of the uppermost part of the water column was higher than that of 1945–1982, when mass mortalities were never observed. The relationship between mortalities and temperature trends at various depths was shown qualitatively by superimposing observed mortalities to temperature trends, but also quantitatively by counting mortalities observed in areas with a negative temperature trend in time vs areas with a positive trend.

In addition, the analysis of *in situ* temperature profiles shows that the Mediterranean Sea is changing in a non-homogeneous fashion (both across the basin and at different depth layers). The areas subjected to mass mortalities correspond to positive thermal anomalies, and the frequency of these co-occurring events is increasing. In the last twenty years, some attempts have been made at ascribing mass mortalities to positive thermal anomalies occurring in the water column [9], [34], [35]. However, evidences are sparse and limited to short time series. Here, we provide for the first time a systematic data collection of all available *in situ* vertical profiles of temperature for the uppermost part of the Mediterranean Sea water column (0–50 m), combined with a review on mass mortalities occurring at basin scale. This allows depicting a picture on the potential effects of temperature changes at Mediterranean scale, never addressed before.

In the literature, clear increases in SST have been repeatedly documented, leading to the conclusion that the surface layer of the Mediterranean Sea is warming [24], [25]. However, when the analysis is extended to the vertical structure of temperature in the upper part of the water column across the basin, a more complex picture emerges.

Irregular distribution in time, spatial inhomogeneity, and change of instrumentation add noise to the *in situ* data, possibly resulting in data over-dispersal, increasing the potential of masking small mean temperature changes and, possibly, also general trends. The used instrumentation (bottles, XBTs, MBTs, CTDs), and their possible malfunctioning are presently debated in the scientific literature [58]–[60]. Systematic errors in depth estimation using XBTs produce biases in temperatures that are difficult to compensate without an independent reliable dataset. In addition, the high number of mass mortality events observed during the last decades might also be a reflection of the more intense observation efforts compared to the earlier period (before 1983). Future, dedicated observation is essential for reaching robust conclusions.

Some of the areas featured by thermal anomalies (both positive and negative) in our study coincide with the areas identified by Coll et al. [23] and by Micheli et al. [21] as prioritary for future conservation and management actions. Warming trends are present in the gulf of Lyons, some portions of the Adriatic Sea, the Tyrrhenian Sea, and from the Ionian Sea until the Nile Delta, overlapping with areas where other cumulative impacts insist, stressing the need for systematic research and management actions.

The observed modifications in thermal conditions affect biodiversity distribution. Increases in the abundance of thermo-tolerant species, [61]–[64], disappearance or rarefaction and mass mortality events of ‘cold’ stenothermal species have all been attributed, directly or indirectly, to temperature changes in the upper layer of the water column [17], [20], [65]. Our results strongly support the hypothesis that mass mortalities are mostly driven by temperature changes. At basin scale, these events were consistently documented where increased temperature trends were registered. At local scale, in those years and months when mass mortalities occurred, almost all thermal profiles show temperatures higher than the average profile calculated for the period 1945–1982 and, even more important, than the average profile calculated for the period 1983–2011.

Oceanographers have been so far cautious when dealing with Mediterranean warming [29], [32], whereas marine ecologists give it for granted [17], [35] substantiating this process from changes in ecological systems and mostly referring to surface and shallow-water temperatures. As shown from our review on mass mortalities, there is increasing evidence of modifications in organisms' distribution. In general, climate warming drive species ranges northwards in the Northern Hemisphere and southwards in the Southern Hemisphere [66], [8] and this tendency is broadly confirmed in the Mediterranean realm [65]. This phenomenon has been named “meridionalization” [67]–[69], since “meridional” species, typical of the southern and usually warmer sectors of the Mediterranean basin, are spreading northwards. More than 30 Mediterranean warm-water indigenous fish species have now been recorded North of their original geographical distribution. For some of these fishes, similar pole-ward extensions have been also recorded in extra-Mediterranean areas, thus reinforcing the consistency of this pattern [20]. Similar range extensions have been recorded for sedentary organisms and benthic macro-algae [65], [70], [71]. Generally, an increase in species richness ensues from climate warming [72]. The timing of the records of species of Mediterranean hydrozoa shows that cold water species tend to be increasingly less recorded than warm water ones [73]. In the Northern Adriatic, zooplankton relic cold-water species such as *Pseudocalanus elongatus* are restricting their winter appearance due to fall temperature increase (see Fonda Umani and Conversi [74]). In Tunisia, the distribution area of the mussel *Mytilus galloprovincialis* is increasingly restricted toward colder areas, less influenced by sea warming [20]. Since slow-growing, benthic suspension feeders efficiently extract and process energy from planktonic ecosystems [75], mortalities affecting this functional group may induce long-term effects on both planktonic and benthic communities. In this situation, the biota represent a reliable proxy for ecological responses to global change.

Our analysis suggests that signature of global warming in the surface layers of the Mediterranean is emerging at basin scale. Models of climate change predict an increase in the probability of occurrence of extreme meteorological events [76]–[79], affecting more the temporal variation than the mean intensity of events over ecological time [80]. Our analysis shows that strong temperature fluctuations determining spatially variable thermal anomalies in the upper layer of the water column (0–50 m) might precede a general, consistent change at basin scale. Such change could be neither gradual nor homogeneous in space and time but, rather, could face a period of increased variability with an increasing number of local episodes of warming before changes embrace the whole basin, as predicted by models [81].

Our results encourage mitigation initiatives. Different oceanographic conditions characterize the basin, possibly allowing for a regional management of climate change effects, based on the identification of consistent conservation units. The establishment of networks of Marine Protected Areas can cope with large-scale environmental impacts, including global climate change, on ocean ecosystems [82]. Enhanced local resistance and resilience by removal or decrease of local disturbances may help combat the effects of global impacts [21]. Implementing monitoring programs to understand the magnitude of climate change, together with specific conservation actions, should be the core of future management strategies for the whole Mediterranean Sea [83].

## Supporting Information

S1 Fig **Subdivision of the Mediterranean Sea into 11 sub-basins according to its broad-scale circulation.**
(DOC)

S2 Fig **Temporal distribution of the number of temperature profiles.**
(DOCX)

S3 Fig **Spatial distribution of the number of temperature profiles.**
(DOC)

S1 Table **Table reporting all documented mass mortality events across the Mediterranean Sea from 1983 to 2009. n.r. =  not reported.**
(XLSX)

S2 Table **Observed mortalities** ***vs*** **temperature trend intervals, along with the results of the Kolmogorov-Smirnov test.**
(DOCX)

S3 Table **Frequency of warm temperatures.**
(DOC)

S1 Appendix **Quality control procedure used to check temperature profiles.**
(DOC)

S2 Appendix **References cited in supplementary** **S1 Table** **.**
(DOC)
