# Supplementary material for: Global Warming and Mass Mortalities of Benthic Invertebrates in the Mediterranean Sea
Source: PLoS One. 2014 Dec 23;9(12):e115655. doi: 10.1371/journal.pone.0115655 (PMC4275269; doi:10.1371/journal.pone.0115655)
Supplement: S1 Fig — Subdivision of the Mediterranean Sea into 11 sub-basins according to its broad-scale circulation. (DOC) [file pone.0115655.s001.doc]

**Figure S1** **Subdivision of the Mediterranean Sea into 11 sub-basins according to its broad-scale circulation.** Sub-basins exceeding 2,000 m bottom depth (Liguro-Provençal, Algerian, Tyrrhenian, Ionian, Cretan and Levantine sub-basins) have been further divided into two areas, a part deeper and a part shallower than 2,000 m, denoted with two different colors. The Adriatic Sea was divided using a different shallow water threshold (250 m).
