# Supplementary material for: Global Warming and Mass Mortalities of Benthic Invertebrates in the Mediterranean Sea
Source: PLoS One. 2014 Dec 23;9(12):e115655. doi: 10.1371/journal.pone.0115655 (PMC4275269; doi:10.1371/journal.pone.0115655)
Supplement: S2 Fig — Temporal distribution of the number of temperature profiles. (DOCX) [file pone.0115655.s002.docx]

**Figure S2** **Temporal distribution of the number of temperature profiles.** Number of temperature profiles in the Mediterranean Sea for year and for the months July-November. Temperature profiles were collected using reversing thermometers attached to bottles, Conductivity-Temperature-Depth (CTD) measuring instruments, Mechanical Bathy-Thermographs (MBT) and eXpendable Bathy-Thermographs (XBT). Colors denote different instruments used for temperature data collection.
