# Supplementary material for: Global Warming and Mass Mortalities of Benthic Invertebrates in the Mediterranean Sea
Source: PLoS One. 2014 Dec 23;9(12):e115655. doi: 10.1371/journal.pone.0115655 (PMC4275269; doi:10.1371/journal.pone.0115655)
Supplement: S3 Fig — Spatial distribution of the number of temperature profiles. (DOC) [file pone.0115655.s003.doc]

**Figure S3 Spatial distribution of the number of temperature profiles.** Colors show the number of observations in each 0.5°lat x 0.5°lon cell for the period 1945-2011 in July (Jul), August (Aug), September (Sep), October (Oct) and November (Nov).
