# Supplementary material for: Global Warming and Mass Mortalities of Benthic Invertebrates in the Mediterranean Sea
Source: PLoS One. 2014 Dec 23;9(12):e115655. doi: 10.1371/journal.pone.0115655 (PMC4275269; doi:10.1371/journal.pone.0115655)
Supplement: S2 Table — Observed mortalities vs temperature trend intervals, along with the results of the Kolmogorov-Smirnov test. (DOCX) [file pone.0115655.s005.docx]

**Table S2 Observed mortalities *vs* temperature trend intervals, along with the results of the Kolmogorov-Smirnov test.**

| temperature trend  interval | > -0.03  ≤ - 0.012  °C/yr | > -0.012  ≤ 0.006  °C/yr | > 0.006  ≤ 0.024  °C/yr | > 0.024  ≤ 0.042  °C/yr | > 0.042  ≤ 0.06  °C/yr | K-S test  statistical  significance |
| --- | --- | --- | --- | --- | --- | --- |
| **observed**  **mortalities** | **3** | **0** | **1** | **14** | **15** | **> 99%** |

The expected mortalities are calculated according to a uniform distribution with respect to the temperature trend. Results are relative to areas where the temperature trend was observed with a 90% statistical significance. The test null hypothesis is the independence of the distribution of mortalities from the temperature trend, which can be rejected with a probability larger than 99%.
