# Supplementary material for: Global Warming and Mass Mortalities of Benthic Invertebrates in the Mediterranean Sea
Source: PLoS One. 2014 Dec 23;9(12):e115655. doi: 10.1371/journal.pone.0115655 (PMC4275269; doi:10.1371/journal.pone.0115655)
Supplement: S3 Table — Frequency of warm temperatures. (DOC) [file pone.0115655.s006.doc]

**Table S3** **Frequency of warm temperatures.**

| **Areas** | **Depth layer** | **Month** | **Freq T>mean T** | **Freq T>mean T+std** | **Freq T>mean T** | **Freq T>mean T+std** |
| --- | --- | --- | --- | --- | --- | --- |
|  |  |  | **(1945-1982)** | **(1945-1982)** | **(1983-2011)** | **(1983-2011)** |
| **Ligurian Sea** | 0-10 m | 7 | 0.37 | 0.15 | 0.07 | 0.04 |
|  | 11-30 m | 7 | 0.33 | 0.04 | 0.22 | 0.04 |
|  | 31-50 m | 7 | 0.26 | 0.07 | 0.15 | 0.07 |
|  | 0-10 m | 8 | 0.43 | 0.10 | 0.14 | 0.00 |
|  | 11-30 m | 8 | 0.38 | 0.19 | 0.10 | 0.00 |
|  | 31-50 m | 8 | 0.38 | 0.19 | 0.05 | 0.00 |
|  | 0-10 m | 9 | 0.18 | 0.04 | 0.36 | 0.14 |
|  | 11-30 m | 9 | 0.18 | 0.04 | 0.29 | 0.21 |
|  | 31-50 m | 9 | 0.18 | 0.04 | 0.25 | 0.11 |
|  | 0-10 m | 10 | 0.31 | 0.06 | 0.29 | 0.11 |
|  | 11-30 m | 10 | 0.29 | 0.06 | 0.29 | 0.14 |
|  | 31-50 m | 10 | 0.31 | 0.03 | 0.20 | 0.09 |
|  | 0-10 m | 11 | 0.19 | 0.03 | 0.29 | 0.16 |
|  | 11-30 m | 11 | 0.16 | 0.03 | 0.29 | 0.16 |
|  | 31-50 m | 11 | 0.23 | 0.03 | 0.29 | 0.16 |
|  |  |  |  |  |  |  |
| **Provence Coast** | 0-10 m | 7 | 0.23 | 0.11 | 0.26 | 0.11 |
|  | 11-30 m | 7 | 0.34 | 0.08 | 0.19 | 0.08 |
|  | 31-50 m | 7 | 0.34 | 0.05 | 0.18 | 0.11 |
|  | 0-10 m | 8 | 0.25 | 0.04 | 0.25 | 0.12 |
|  | 11-30 m | 8 | 0.27 | 0.06 | 0.24 | 0.08 |
|  | 31-50 m | 8 | 0.43 | 0.10 | 0.12 | 0.06 |
|  | 0-10 m | 9 | 0.27 | 0.05 | 0.29 | 0.08 |
|  | 11-30 m | 9 | 0.24 | 0.08 | 0.26 | 0.08 |
|  | 31-50 m | 9 | 0.26 | 0.05 | 0.23 | 0.06 |
|  | 0-10 m | 10 | 0.29 | 0.05 | 0.19 | 0.09 |
|  | 11-30 m | 10 | 0.33 | 0.07 | 0.19 | 0.07 |
|  | 31-50 m | 10 | 0.36 | 0.07 | 0.17 | 0.05 |
|  | 0-10 m | 11 | 0.21 | 0.04 | 0.27 | 0.15 |
|  | 11-30 m | 11 | 0.23 | 0.04 | 0.27 | 0.15 |
|  | 31-50 m | 11 | 0.27 | 0.04 | 0.19 | 0.10 |
|  |  |  |  |  |  |  |
| **Eastern Tyrrhenian** | 0-10 m | 7 | 0.29 | 0.10 | 0.21 | 0.10 |
|  | 11-30 m | 7 | 0.29 | 0.02 | 0.19 | 0.13 |
|  | 31-50 m | 7 | 0.29 | 0.04 | 0.19 | 0.08 |
|  | 0-10 m | 8 | 0.22 | 0.09 | 0.24 | 0.09 |
|  | 11-30 m | 8 | 0.39 | 0.04 | 0.17 | 0.11 |
|  | 31-50 m | 8 | 0.41 | 0.07 | 0.15 | 0.04 |
|  | 0-10 m | 9 | 0.28 | 0.06 | 0.22 | 0.08 |
|  | 11-30 m | 9 | 0.24 | 0.04 | 0.28 | 0.14 |
|  | 31-50 m | 9 | 0.20 | 0.06 | 0.18 | 0.10 |
|  | 0-10 m | 10 | 0.23 | 0.04 | 0.19 | 0.11 |
|  | 11-30 m | 10 | 0.21 | 0.06 | 0.23 | 0.09 |
|  | 31-50 m | 10 | 0.28 | 0.02 | 0.23 | 0.09 |
|  | 0-10 m | 11 | 0.28 | 0.08 | 0.24 | 0.10 |
|  | 11-30 m | 11 | 0.28 | 0.06 | 0.22 | 0.10 |
|  | 31-50 m | 11 | 0.30 | 0.06 | 0.20 | 0.08 |
|  |  |  |  |  |  |  |
| **Western Tyrrhenian** | 0-10 m | 7 | 0.34 | 0.11 | 0.17 | 0.03 |
|  | 11-30 m | 7 | 0.37 | 0.06 | 0.17 | 0.11 |
|  | 31-50 m | 7 | 0.23 | 0.03 | 0.20 | 0.09 |
|  | 0-10 m | 8 | 0.32 | 0.07 | 0.11 | 0.11 |
|  | 11-30 m | 8 | 0.43 | 0.18 | 0.11 | 0.00 |
|  | 31-50 m | 8 | 0.46 | 0.18 | 0.00 | 0.00 |
|  | 0-10 m | 9 | 0.24 | 0.07 | 0.26 | 0.07 |
|  | 11-30 m | 9 | 0.26 | 0.07 | 0.19 | 0.10 |
|  | 31-50 m | 9 | 0.24 | 0.12 | 0.14 | 0.05 |
|  | 0-10 m | 10 | 0.33 | 0.17 | 0.10 | 0.07 |
|  | 11-30 m | 10 | 0.33 | 0.07 | 0.13 | 0.00 |
|  | 31-50 m | 10 | 0.40 | 0.10 | 0.07 | 0.00 |
|  | 0-10 m | 11 | 0.25 | 0.14 | 0.22 | 0.06 |
|  | 11-30 m | 11 | 0.28 | 0.14 | 0.17 | 0.08 |
|  | 31-50 m | 11 | 0.33 | 0.08 | 0.11 | 0.06 |
|  |  |  |  |  |  |  |
| **Balearic and Columbretes Islands** | 0-10 m | 7 | 0.18 | 0.03 | 0.28 | 0.08 |
|  | 11-30 m | 7 | 0.28 | 0.03 | 0.25 | 0.10 |
|  | 31-50 m | 7 | 0.35 | 0.05 | 0.20 | 0.05 |
|  | 0-10 m | 8 | 0.21 | 0.03 | 0.33 | 0.05 |
|  | 11-30 m | 8 | 0.21 | 0.03 | 0.31 | 0.18 |
|  | 31-50 m | 8 | 0.23 | 0.03 | 0.26 | 0.05 |
|  | 0-10 m | 9 | 0.28 | 0.05 | 0.23 | 0.10 |
|  | 11-30 m | 9 | 0.33 | 0.03 | 0.26 | 0.05 |
|  | 31-50 m | 9 | 0.26 | 0.10 | 0.21 | 0.05 |
|  | 0-10 m | 10 | 0.33 | 0.04 | 0.22 | 0.11 |
|  | 11-30 m | 10 | 0.26 | 0.07 | 0.24 | 0.11 |
|  | 31-50 m | 10 | 0.33 | 0.07 | 0.20 | 0.07 |
|  | 0-10 m | 11 | 0.22 | 0.02 | 0.33 | 0.13 |
|  | 11-30 m | 11 | 0.20 | 0.02 | 0.33 | 0.13 |
|  | 31-50 m | 11 | 0.27 | 0.09 | 0.29 | 0.09 |

For each areas the monthly frequency of temperatures beyond the mean temperature and beyond the mean temperature + standard deviation calculated for the period 1945-2011, is recorded for depth layer and month, corresponding to the periods 1945-1982 and 1992-2011.
