# Supplementary material for: Global Warming and Mass Mortalities of Benthic Invertebrates in the Mediterranean Sea
Source: PLoS One. 2014 Dec 23;9(12):e115655. doi: 10.1371/journal.pone.0115655 (PMC4275269; doi:10.1371/journal.pone.0115655)
Supplement: S1 Appendix — Quality control procedure used to check temperature profiles. (DOC) [file pone.0115655.s007.doc]

**Appendix S1 Quality control procedure used to check temperature profiles.**

The quality control procedure adopted for the temperature profiles used in this study consisted of the following steps:

1. only valid data, according to the quality flag definition [1,2], have been kept;
2. temperature data outside the range 5°C-35°C have been excluded;
3. temperature profiles starting at a depth greater than 10 m or terminating at a depth lower than 50 m have been removed;
4. spikes have been removed following the procedure described by Manzella et al. [3];
5. profiles with less than 7 valid data have been removed;
6. in general outliers have been removed.

For the identification of outliers the Mediterranean has been divided into 11 sub-basins, following the subdivision used by MEDAR/MEDATLAS. All sub-basins were further divided into a part deeper than 2,000 m and a part shallower than 2,000 m. The Strait of Sicily, the Aegean Sea and the Alboran Sea contain no part deeper than 2,000 m. The Adriatic Sea was divided using a shallow 250 m threshold (Fig. S1). For each month and for each sub-basin subdivision in respect to the bottom depth, all profiles have been plotted and profiles with a structure significantly different from the adjacent ones have been eliminated.

**References**

1. Fichaut M, Garcia MJ, Giorgetti A, Iona A, Kushmaro A, et al. (2003) MEDAR/ MEDATLAS 2002: a Mediterranean and Black Sea database for the operational Oceanography. In: Dahlin H, et al., editors. Building the European capacity in operational oceanography: proceedings of the 3rd International Conference on EuroGOOS 3-6 December, 2002, Athens, Greece. Elsevier Oceanography Series, 69. pp. 645-648.
2. Johnson DR, Garcia HE, Boyer TP (2013) World Ocean Database 2013 Tutorial. Sydney Levitus, Ed.; Alexey Mishonov, Technical Ed.; NODC Internal Report 23, NOAA Printing Office, Silver Spring, MD. 25 p.

Available at http://www.nodc.noaa.gov/OC5/WOD13/docwod13.html.

1. Manzella GMR, Scoccimarro E, Pinardi N and Tonani M (2003) Improved near real-time data management procedures for the Mediterranean ocean forecasting System-Voluntary Observing Ship program. Ann Geophys 21: 49-62.
