# Supplementary material for: Global Warming and Mass Mortalities of Benthic Invertebrates in the Mediterranean Sea
Source: PLoS One. 2014 Dec 23;9(12):e115655. doi: 10.1371/journal.pone.0115655 (PMC4275269; doi:10.1371/journal.pone.0115655)
Supplement: S2 Appendix — References cited in supplementary S1 Table . (DOC) [file pone.0115655.s008.doc]

**Appendix S2 References cited in supplementary Table S1.**

1. Harmelin, JG (1984) Biologie du Corail Rouge. Parametres de populations, croissance et mortalite. Etat des connaissances en France. In: Charbonnier D, Garcia S, editors. Rapport de Consultation Technique du CGPM sur les Ressources du Corail Rouge de la Mediterranee Occidentale et Leur Exploitation Rationelle (FAO Rapport 306 sur les Peches). FAO, Palma de Mallorca, Spain. pp. 99–103.
2. Bavestrello G, Boero F (1988) Necrosi e rigenerazione di *Eunicella cavolinii* in Mar Ligure. Bollettino dei Musei e degli Istituti Biologici dell’Università di Genova 52: 295-300.
3. Gaino E, Pronzato R (1989) Ultrastructural evidence of bacterial damage to *Spongia officinalis* fibres (Porifera, Demospongiae). Dis Aquat Organ 6: 67-74.
4. Gaino E, Pronzato R, Corriero G, Buffa P (1992) Mortality of commercial sponges: incidence in two Mediterranean areas. Ital J Zool 59: 79-85.
5. Coma R, Zabala M (1992) Seguiment temporal de la gorgonia *Paramuricea clavata* de les illes Medes. Exercici 1992. Seguiment temporal de les Illes Medes. Informe anual 1992. Departament d’Agricultura, Ramaderia i Pesca, Generalitat de Catalunya, Barcelona, Spain.
6. Mistri M, Ceccherelli VU (1996) Effects of a mucilage event on the Mediterranean gorgonian *Paramuricea clavata* I‐Short term impacts at the population and colony levels. Ital J Zool 63: 221-230.
7. Bavestrello G, Bertone S, Cattaneo-Vietti R, Cerrano C, Gaino E, et al. (1995) Mass mortality of *Paramuricea clavata* (Anthozoa, Cnidaria) on Portofino Promontory cliffs, Ligurian Sea, Mediterranean Sea. Mar Life 4: 15-19.
8. Harmelin JC, Marinopoulos J (1994) Population structure and partial mortality of the gorgonian *Paramuricea clavata* (Risso) in the North-Western Mediterranean (France, Port-Cros Island). Mar Life 4: 5-13.
9. Corriero G, Scalera-Liaci L, Rizzello R. (1996) Osservazioni sulla mortalita di *Ircinia spinosula* (Schmidt) and *Ircinia* sp. (Porifera, Demospongiae) nell'insenatura della Strea di Porto Cesareo. Thalassia Salent 22: 51–62
10. Rodolfo-Metalpa R, Bianchi CN, Peirano A and Morri C (2000) Coral mortality in NW Mediterranean. Coral Reefs 19: 24.
11. Rodolfo-Metalpa R, Bianchi CN, Peirano A and Morri C (2005) Tissue necrosis and mortality of the temperate coral *Cladocora caespitosa*. Ital J Zool 72: 271-276.
12. Cerrano C, Magnino G, Sara A, Bavestrello G, Gaino E (2001) Necrosis in a population of *Petrosia ficiformis* (Porifera, Demospongiae) in relation with environmental stress. Ital J Zool 68: 131-136.
13. Cerrano C, Bavestrello G, Bianchi CN, Cattaneo-vietti R, Bava S, et al. (2000) A catastrophic mass-mortality episode of gorgonians and other organisms in the Ligurian Sea (Northwestern Mediterranean), summer 1999. Ecol Lett 3: 284-293.
14. Cupido R, Cocito S, Sgorbini S, Bordone A and Santangelo G (2008) Response of a gorgonian (*Paramuricea clavata*) population to mortality events: recovery or loss? Aquat Conserv 18: 984-992.
15. Perez T, Garrabou J, Sartoretto S, Harmelin JG, Francour P, et al. (2000) Mass mortality of marine invertebrates: an unprecedented event in the Northwestern Mediterranean. Comptes Rendus De L Academie Des Sciences Serie III-Sciences De La Vie-Life Sciences 323: 853-865.
16. Linares C, Coma R, Diaz D, Zabala M, Hereu B, et al. (2005) Immediate and delayed effects of a mass mortality event on gorgonian population dynamics and benthic community structure in the NW Mediterranean Sea. Mar Ecol Prog Ser 305: 127-137.
17. Linares C, Coma R, Zabala M (2008) Effects of a mass mortality event on gorgonian reproduction. Coral Reefs 27: 27-34.
18. Garrabou J, Perez T, Sartoretto S, Harmelin JG (2001) Mass mortality event in red coral *Corallium rubrum* populations in the Provence region (France, NW Mediterranean). Mar Ecol Prog Ser 217: 263-272.
19. Coma R, Linares C, Ribes M, Diaz D, Garrabou J, et al. (2006) Consequences of a mass mortality in populations of *Eunicella singularis* (Cnidaria: Octocorallia) in Menorca (NW Mediterranean). Mar Ecol Prog Ser 327: 51-60.
20. Bensoussan N, Romano JC, Harmelin JG, Garrabou J (2010) High resolution characterization of northwest Mediterranean coastal waters thermal regimes: to better understand responses of benthic communities to climate change. Estuar Coast Shelf S 87: 431-441.
21. Cerrano C, Totti C, Sponga F, Bavestrello G (2006) Summer disease in *Parazoanthus axinellae* (Schmidt, 1862) (Cnidaria, Zoanthidea). Ital J Zool 73: 355-361.
22. Calvisi G, Trainito E, Pais MM, Franci G, Schiapparelli S (2003) Prima segnalazione di un evento di mortalità di Gorgonacei lungo la costa dell’isola di Tavolaria (Sardegna settentrionale). Biol Mar Mediterr 10(2): 506-508.
23. Gambi MC, Barbieri F, Signorelli S, Saggiomo V (2010) Mortality events along the Campania coast (Tyrrhenian Sea) in summers 2008 and 2009 and relation to thermal conditions. Biol Mar Mediterr 17(1): 126-127.
24. Garrabou J, Coma R, Bensoussan N, Bally M, Chevaldonne P, et al. (2009) Mass mortality in Northwestern Mediterranean rocky benthic communities: effects of the 2003 heat wave. Global Change Biol 15: 1090-1103.
25. Bally M, Garrabou J (2007) Thermodependent bacterial pathogens and mass mortalities in temperate benthic communities: a new case of emerging disease linked to climate change. Global Change Biol 13: 2078-2088.
26. Kersting D-K, García-March JR, Templado J (2006) Evaluation of *Spondylus gaederopus* Linneo, 1758 mass mortality event in the Columbretes Islands Marine Reserve (Western Mediterranean, Spain). International Congress on Bivalvia UAB.
27. Cigliano M, Gambi MC (2007) The long hot summer: a further mortality event of gorgonians along the Phlaegrean Islands (Tyrrhenian Sea). Biol Mar Mediterr 14: 292-293.
28. Vezzulli L, Previati M, Pruzzo C, Marchese A, Bourne DG, et al. (2010) Vibrio infections triggering mass mortality events in a warming Mediterranean Sea. Environ Microbiol 12: 2007-2019.
29. Calvo E, Serrano E, Linares C (2007) Informe sobre el estado de la poblaciones de gorgonias de la reserva marina de las Islas Hormigas (Servicio de Pesca y Acuicultura de la Comunidad Autónoma de Murcia).
30. Huete-Stauffer C, Vielmini I, Palma M, Navone A, Panzalis P, et al. (2011) *Paramuricea clavata* (Anthozoa, Octocorallia) loss in the Marine Protected Area of Tavolaria (Sardinia, Italy) due to a mass mortality event. Marine Ecology 32: 107-116.
31. Cebrian E, Jesus Uriz M, Garrabou J, Ballesteros E (2011) Sponge Mass Mortalities in a Warming Mediterranean Sea: Are Cyanobacteria-Harboring Species Worse Off? Plos One 6.
32. Maldonado M, Sànchez-Tocino L, Navarro C (2010) Recurrent disease outbreaks in corneous demosponges of the genus *Ircinia*: epidemic incidence and defense mechanisms. Mar biol 157: 1577-1590.
33. Gambi MC, Cigliano M, Iacono B (2006) Mortality events along the Campania coast (Tyrrhenian Sea) in summers 2008 and 2009 and relation to thermal conditions. Biol Mar Mediterr 17: 126-127.
34. Stabili L, Cardone F, Alifano P, Tredici SM, Piraino S, et al. (2012) Epidemic mortality of the sponge *Ircinia variabilis* (Schmidt, 1862) associated to proliferation of a *Vibrio* bacterium. Microb Ecol 64: 802-813.
35. Di Camillo CG, Bartolucci I, Cerrano C, Bavestrello G (2013) Sponge disease in the Adriatic Sea. Marine Ecology 34: 62-71.
